# Comparison of frequencies of non motor symptoms in Indian Parkinson’s disease patients on medical management versus deep brain stimulation: A case-control study

**Published:** 2015-04-04

**Authors:** Kandadai Rukmini Mridula, Rupam Borgohain, Shaik Afshan Jabeen, Gaddamanugu Padmaja, VCS Srinivasarao Bandaru, Praveen Ankathi, Meena A Kanikannan, Mohammed Shujath Ali Khan

**Affiliations:** 1 Department of Neurology, Nizam’s Institute of Medical Sciences, Autonomous University, Hyderabad, India; 2 Department of Research, Yashoda Hospital Somajiguda Hyderabad, India; 3 Department of Neurosurgery, Nizam’s Institute of Medical Sciences, Autonomous University, Hyderabad, India

**Keywords:** Parkinson Disease, Deep Brain Stimulation, Subthalamic Nucleus, Dopamine Agents, Comprehensive Health Care

## Abstract

**Background: **Non motor symptoms (NMS) of idiopathic Parkinson’s disease (PD) are a major cause of disability and recognition of these symptoms and treatment is important for comprehensive health care. Deep brain stimulation of bilateral subthalamic nucleus deep brain stimulation (STN DBS) has been shown to improve motor symptoms in PD and effects on NMS are unknown. To investigate the NMS among PD patients who underwent STN DBS.

**Methods:** We recruited prospectively 56 patients with PD, who had undergone bilateral STN DBS and 53 age and duration of illness matched PD patients on dopaminergic therapy (controls). NMS were assessed using 30 item questionnaire NMS Quest. These questions evaluated 9 domains, gastrointestinal, urinary, cardiovascular, sexual, cognition (apathy/attention/memory), anxiety/depression, hallucinations/delusions, sleep and miscellaneous. Comparison was done on individual symptoms as well as in various domains. This study was carried at Nizam’s Institution of Medical Sciences and study period was from January 2011 to December 2012.

**Results: **Patients who underwent STN DBS had a significantly lower mean total score on NMS quest (6.7 ± 3.8) compared to controls (8.4 ± 3.7) (P < 0.00100). Symptoms in the domains of cardiovascular, gastrointestinal, sleep were significantly less frequent while sexual disturbances were significantly more frequent among patients compared to controls. On individual symptom analysis, nocturia  (P < 0.00010), unexplained pains (P < 0.00010), nausea and vomiting, constipation, lightheadedness, depression, and insomnia were less prevalent, while sexual disturbances were significantly more common in STN DBS group compared to controls.

**Conclusion: **Bilateral STN DBS not only improves the motor symptoms but also improves many NMS in PD patients.

## Introduction

Parkinson’s disease (PD) is the second most common neurodegenerative worldwide,^1^ with a prevalence of 52.85/100,00 in India.^2^ The emphasis in most of the last decade has been on the motor symptoms of PD, concentrating mainly on tremor, rigidity, postural instability, and bradykinesia. It is now increasingly recognized that the disease is more pervasive with various non motor manifestations. Non motor symptoms (NMS) of PD are common in all stages of the disease, are very often under recognized^3^ and are a major cause of disability.^4^ Recognizing and treating these symptoms are essential for improving functional outcome. Deep brain stimulation (DBS) of the bilateral subthalamic nucleus (STN) or globus pallidus has been established to be superior to oral dopaminergic medications for control of motor symptoms.^5^ However, there are very few studies which estimated the effect of DBS on NMS.^6^^,^^7^ The objective of the present study was to investigate the NMS in PD patients who have undergone bilateral STN DBS. Limited data are available from the Indian subcontinent.

## Materials and Methods

We recruited prospectively 56 cases and 53 controls from movement disorder clinic at Nizam’s Institution of Medical Sciences (NIMS) Hyderabad India. NIMS is one of the referral university teaching hospitals in South India. We used United Kingdom PD society brain bank criteria^8^ for diagnosis of PD in both cases and controls. This study was approved by the Institutional ethical committee and study period from January 2011 to December 2012.

Cases are defined patients who underwent bilateral STN DBS with ≥ 1 year follow-up were considered as cases. Similar age and duration of illness matched 53 PD patients on oral dopaminergic therapy was defined as controls.

Inclusion criteria were PD disease duration of ≥6 years, good response to levodopa (improvement in Unified PD Rating Scale [UPDRS] part III by more than 30%), able to walk independently in drug “on” state (Hoehn and Yahr stage < 4 in “on” state), normal cognition (Montreal cognitive assessment > 25). Both case and control patients who were wheelchair or bed bound had dementia, or severe psychiatric disturbances were excluded.

Detailed neurological examination was done in case controls by movement disorder specialist and neurologist. Both cases and control’s present and past medical records were reviewed by a trained neurology resident. All patients were on appropriate pharmacological and non-pharmacological therapies (e.g., physical, occupational, and speech therapies) titrated to achieve optimal functioning.

Assessment of motor deficits was done in both “off” (dopaminergic drugs stopped for a period of 12 h) and “on” states (after the patient has the maximum improvement with medication), using UPDRS part III which evaluates the motor functions. Among cases, the neurostimulator was kept “on” and thus the “off” state was in “medication off, stimulator on” state while “on” state was in “medication on stimulator on” state.^9^

NMS were assessed using NMS Quest, a 30 item comprehensive questionnaire which assesses all NMS. All items detect the presence or absence of symptoms based on yes-no answers.^10^ The questionnaire was taken from the patient in the “on” state (stimulator “on” and medication “on” in cases and medication “on” in controls) by a movement disorder specialist. We further classified the 30 questions in 9 domains: gastrointestinal, urinary, cardiovascular, sexual, cognition (apathy/attention/memory), anxiety/depression, hallucinations/delusions, sleep and miscellaneous.^4^ Seven questions i.e., dribbling of saliva, reduced taste or smell, dysphagia, nausea, constipation, bowel incontinence and incomplete bowel emptying were included in gastrointestinal domain. Cardiovascular domain included 2 questions–feeling light-headed and falling (syncope) while urinary domain included questions on urgency and frequency of micturition. Memory problems, loss of interest and difficulty in concentration were classified under memory domain while feeling sad and feeling anxious/frightened were questions in anxiety/depression domain. Presence of hallucinations and delusions were the two questions in hallucinations/depression domain while reduced interest and difficulty in performing sex were included in a sexual domain. Sleep domain included 5 questions on insomnia, increased drowsiness with difficulty in staying awake, vivid dreams, talking or moving in sleep (rem sleep behavioral disorders), unpleasant sensations in leg (restless leg syndrome) whereas the last 5 questions on unexplained pains, changes in weight, swelling of feet, excessive sweating, and double vision were included in miscellaneous domain.^4^

The frequency of involvement in each domain was further analyzed and compared among the two groups.

The detailed medication history was noted from both cases and controls. This included the dosage of various dopaminergic drugs levodopa-carbidopa combination, dopaminergic agonists (pramipexole, ropinirole), amantadine, trihexyphenidyl (anticholinergics) and monoamine oxidase B inhibitors (rasagiline and selegiline).

Levodopa equivalent daily dosage (LEDD) was calculated for each patient to finally calculate the total dose.^11^

Continuous variables were presented in titer of mean and ± standard deviation. Student t-test was used to study the difference between the two groups. Categorical variables were expressed as proportions, and chi-square test was used to study the difference between two groups. The medication in each group was analyzed based on the percentage of patients on each drug as well as the mean LEDD. Odds ratio was used to assess the impact of DBS on NMS. All tests were two-sided and P < 0.05000 was considered statistically significant.

## Results

Mean age of the cases and controls were 57.1 ± 9.4 and 56.6 ± 8.2 years respectively. Men in DBS group and controls constituted 67.8% (38/56) and 81.1% (43/53), respectively. Mean disease duration was 9.39 ± 2.3 and 9.17 ± 2.9 among cases and controls, respectively. The mean duration after surgery in DBS patients was 1.9 ± 2.4 years. On evaluation of UPDRS-III in off state, significantly lower scores among DBS group (UPDRS III in off state 28.8 ± 8.4) compared to controls (UPDRS III in off state 43.2 ± 7.9, P < 0.01000) were detected (Table 1).

The medication in both the groups varied. The levodopa equivalent dose was significantly lower in cases compared to patients on medical management. There was also significantly lesser usage of amantadine in cases. Compared to controls, mean levodopa dosage was lower while the mean dose of pramipexole was significantly higher in cases. This is because of the policy in our institute to manage most patients on dopaminergic agonists predominantly after DBS surgery. Very few patients in both groups were on anticholinergic medication (Table 2).

Overall, 99% of all 109 PD patients reported one or more NMS. The average NMS Quest total score was 7.6 ± 4.1 and ranged from 0 to a maximum of 22. The mean total score on NMS quest was significantly lower among patients who had undergone DBS (6.7 ± 3.8) compared to controls (8.4 ± 3.7, P = 0.02000).

On the comparison of both groups, symptoms in the domains of cardiovascular, gastrointestinal, sleep and miscellaneous were significantly less frequent, while sexual disturbances were significantly more frequent among cases (Table 3).

**Table 1:** Baseline characteristics

**Parameters**	**Cases (n = 56)**	**Controls (n = 53)**	**P**
Men	38	43	0.11000
Age range	34-77	39-75	
Duration of disease range	6-15	6-15	
Mean age	57.80 ± 9.60	56.64 ± 8.22	0.77000
Mean disease duration	9.62 ± 2.48	9.16 ± 2.94	0.39000
Mean UPDRS III score in “off” state	32.90 ± 11.40	43.20 ± 7.90	< 0.00010
Mean UPDRS III score in “on” state	8.80 ± 3.70	9.80 ± 5.50	0.27000

UPDRS: Unified PD Rating Scale

**Table 2:** Percentage and mean dose of various dopaminergic drugs used by cases and controls

**Parameters**	**Cases (n = 56)**	**Controls (n = 53)**	**P**
Number of patients on levodopa [n (%)]	53 (95.0)	50 (94.00)	0.94000
Number of patients on dopamine agonists [n (%)]	51 (91.0)	33 (62.00)	0.00035
Number of patients on anticholinergics [n (%)]	6 (10.0)	12 (22.60)	0.09000
Number of patients on amantadine [n (%)]	8 (14.20)	16 (28.57)	0.04500
Number of patients on MAO-inhibitors [n (%)]	4 (7.10)	4 (7.50)	0.93000
Mean levodopa dose (mg/day) (mean ± SD)	353.50 ± 228.00	447.20 ± 241.10	0.03900
Mean pramipexole dose (mg/24hours) (mean ± SD)	4.19 ± 1.53	3.45 ± 1.94	0.00800
Mean Ropinirole dose (mg/24 hours) (mean ± SD)	4.11 ± 0.86	3.88 ± 1.10	0.93000
Mean levodopa equivalent dose (mg/24 h) (mean ± SD)	672.50 ± 302.4	815.80 ± 414.60	0.04000

MAO: Monoamine oxidase; SD: Standard deviation

**Table 3:** Frequency of involvement of various non motor domains in cases and controls

**NMS domains **	**Cases (n = 56) [n (%)] **	**Controls (n = 53) [n (%)]**	**P**
Gastrointestinal complaints	38 (67.86)	50 (94.3)	< 0.00010
Urinary disturbances	41 (73.2)	46 (86.7)	0.08000
Cardiovascular problems	18 (32.14)	33 (62.2)	< 0.01000
Sexual disturbances	29 (51.7)	15 (28.3)	0.01000
Cognitive impairment/apathy	26 (46.4)	17 (32.0)	0.13000
Anxiety/depression	16 (28.5)	37 (69.8)	< 0.01000
Hallucinations/delusions	12 (21.4)	8 (15.0)	0.39000
Sleep disturbance	25 (44.6)	37 (69.8)	0.01000
Miscellaneous	21 (37.5)	44 (83.0)	< 0.01000

NMS: Non motor symptoms

**Table 4:** Frequency of each non motor symptom in cases and controls

**Individual symptoms [n (%)]**	**Cases (n = 56) **	**Controls (n = 53) **	**Odds **	**P**
Gastrointestinal complaints				
Dribbling of saliva during the daytime	17 (30.4)	25 (47.2)	0.49	0.07000
Loss or change in ability to taste or smell	4 (7.1)	4 (7.5)	0.94	0.94000
Difficulty swallowing food or drink	8 (14.3)	11 (20.7)	0.64	0.37000
Feeling of nausea/vomiting	1 (1.8)	6 (11.3)	0.14	0.04000
Constipation	29 (51.7)	38 (71.7)	0.42	0.03000
Bowel incontinence	3 (5.4)	6 (11.32)	0.44	0.26000
Incomplete bowel emptying	11 (19.6)	15 (28.30)	0.62	0.29000
Cardiovascular abnormalities				
Feeling light headed dizzy	7 (12.5)	28 (52.8)	0.13	< 0.00010
Falling	14 (25)	12 (22.6)	1.14	0.77000
Urinary problems				
Urgency of micturition	27 (48.2)	20 (37.7)	1.54	0.80000
Getting up regularly for urine	35 (62.5)	44 (83.0)	0.34	0.02000
Cognitive impairment/apathy				
Memory problems	14 (25.0)	11 (20.7)	1.27	0.60000
Loss of interest	10 (17.8)	8 (15.1)	1.22	0.70000
Difficulty concentration	7 (12.5)	8 (15.1)	0.80	0.69000
Anxiety/depression				
Feeling sad	16 (28.5)	34 (64.1)	0.22	0.00040
Feeling anxious, frightened	17 (30.3)	14 (26.4)	1.21	0.65000
Hallucinations/delusions				
Seeing things-hallucinations	10 (17.8)	5 (9.4)	2.08	0.30000
Believing things-delusions	3 (5.3)	3 (5.6)	0.94	0.70000
Sexual disturbances				
Feeling less interested in sex	25 (41.1)	14 (26.42)	1.94	0.11000
Finding it difficult to perform sex	26 (42.9)	11 (20.75)	2.86	0.01000
Sleep				
Finding it difficult to stay awake	6 (10.7)	7 (13.2)	0.75	0.90000
Difficulty getting to sleep at night	15 (26.7)	29 (54.7)	0.30	0.00500
Vivid dreams	12 (21.4)	19 (35.8)	0.49	0.100000
Talking or moving in sleep	11 (19.6)	4 (7.5)	2.99	0.07000
Unpleasant sensation in legs	5 (8.9)	8 (15.1)	0.55	0.32000
Miscellaneous/ others				
Unexplained pains	18 (32.1)	41 (77.3)	0.13	< 0.00010
Change in weight	9 (16.1)	7 (13.2)	1.25	0.67000
Swelling of legs	9 (16.1)	3 (5.6)	3.19	0.08000
Excessive sweating	9 (16.1)	9 (17)	0.94	0.90000
Double vision	4 (7.1)	1 (1.9)	4.00	0.19000

Individual symptom analysis showed significantly lower frequency of nocturia, unexplained pains, nausea and vomiting, constipation, lightheadedness, depression, and insomnia while sexual disturbances were significantly more common post DBS (Table 4).

## Discussion

This is a comparative study of NMS in PD patients who have undergone bilateral STN DBS versus controls. All controls and 98% of cases in our study had one or more non motor symptom and similar findings were reported by Krishnan et al.^12^ We noted significantly lower mean total score on NMS Quest in cases compared to controls. Our findings were advocated by other studies.^6^^,^^7^^,^^13^

***Individual non motor domains***

The effects of bilateral STN DBS on individual NMS are varied and still unclear. In our study on comparison of both groups, symptoms in the domains of cardiovascular, gastrointestinal, sleep and miscellaneous were significantly less frequent in controls, while sexual disturbances were significantly more frequent among cases with an odds ratio of 2.72. Witjas et al. in his study noted evaluated fluctuations in NMS and found significant improvement in sensory-painful fluctuations, dysautonomia and cognitive functions in 40 patients after bilateral STN DBS,^14^ while Zibetti et al. found improvement only in constipation and sleep in 36 patients, after bilateral STN DBS when compared to presurgery state.^15^

***Gastrointestinal symptoms***

Among PD patients, gastrointestinal system is a common non motor domain to be involved, and constipation followed by dribbling of saliva are the major symptoms.^16^^-^^19^ In our study, gastrointestinal symptoms were significantly lower in cases (67.8%) compared controls (94.3%), and similar finding have been noted by others.^20^ Among the gastrointestinal symptoms, sialorrhea, constipation, and nausea and vomiting were significantly lower among cases compared to controls. Similar findings have been noted in previous studies. While Zibetti et al. found improvement in constipation^15^ and Ciucci et al. found improved deglutition after DBS surgery.^21^ This effect may be secondary to change in medications. Anticholinergics used may worsen constipation and nausea, but, on the other hand, should improve sialorrhea. As all gastrointestinal NMS have improved, a central cause may also be responsible. The proposed central mechanism is that subthalamic nucleus stimulation possibly modulates the brain stem structures involved in controlling gut motility and secretion.^21^

***Cardiovascular***

We established in our study that significantly reduced cardiovascular symptoms were reported by cases 18 (32.14%) compared to controls 33 (62.2%). Postural hypotension can be disabling and in an epidemiologic study, 9.1% of PD patients required such medications to treat orthostatic hypotension.^22^ Symptomatic postural hypotension evaluated by the presence of lightheadedness was significantly lower in the STN DBS group.

This positive effect may be because of the direct effect of neurostimulation or maybe secondary to levodopa dose reduction. However, there has been conflicting reports regarding the impact of bilateral STN DBS on cardiovascular autonomic functions.^23^ Contrary to our findings, Holmberg et al. in a cohort of 11 patients did not find any change in cardiovascular autonomic functions after STN DBS.^24^ Ludwig et al. in their study demonstrated that STN DBS improved cardiovascular autonomic function by levodopa dose reduction but had no direct effect on cardiovascular autonomic functions.^25^

***Sleep disorder***

Sleep disturbances are a general problem in PD patients.^18^^,^^26^ Varanese et al. noted in his study, sleep disturbance in 98% of patients with PD.^18^ In our study we noted symptoms of insomnia were significantly lower in cases compared to controls. These are similar to previous studies which have shown significant improvement in sleep symptoms with STN DBS.^15^^,^^21^^,^^23^^,^^27^ Motor symptoms of rigidity and bradykinesia can lead to sleep fragmentation and poor sleep functions and STN DBS may improve sleep by improving motor functions.^27^

***Urinary symptoms***

Nocturia is a common problem occurring in approximately 60% of PD patients.^28^^,^^29^ In our study when we compared the frequency of all urinary symptoms, no significant difference was noted between cases and controls. However getting up regularly for urine at night or nocturia was significantly lesser in cases, a similar finding was noted by Halim et al.^30^ In previous studies STN DBS has shown to improve bladder symptoms with decreased detrusor hyperreflexia and increased bladder capacity.^31^^,^^32^ It has postulated that the improvement may be mainly due to modulation of bladder afferents and central sensory processing by STN DBS.

***Cognitive impairment***

Mild cognitive impairment is prevalent in 19-38% of PD patients^33^^-^^35^ and these patients have a high risk of developing dementia.^35^^-^^38^ In our study, we noted slightly higher frequency of cognitive impairment among cases (46.4%) compared to controls(32.%) but the difference was not statistically significant. Cognitive impairment has been noted after bilateral STN DBS.^39^^-^^42^ However, Witjas et al. found significant improvement in fluctuations in cognitive functions after bilateral STN DBS.^14^

***Anxiety/depression***

Several studies have estimated that around 16-70% of PD patients suffer from neuropsychiatric problems, including depression, apathy, psychosis, and anxiety.^43^^-^^45^ In our study anxiety or depression was significantly lower in cases compared to controls, similar findings were noted by others.^46^^,^^47^ However, in few studies of PD patients followed up after STN DBS anxiety was shown to be same pre and post operatively.^48^^,^^49^ The limitation of the previous studies was a lack of the control group, and other unknown factors could have influenced the outcome.

***Sexual dysfunction***

Several reports have found sexual dysfunction to be associated with PD.^50^^-^^53^ The prevalence may range from 22% to 68.4%.^54^^-^^56^ In our study, we found significantly higher frequency of sexual impairment in DBS patient compared to controls. This is in contrary to a prior study which showed improvement in sexual well-being in a cohort of 31 patients, 9-12 months after STN DBS.^57^ The beneficial effect may be due to a reduction in dopaminergic medications which may cause erectile dysfunction, premature ejaculation, and reduced libido.^50^^,^^52^^,^^58^^,^^59^ Compulsive sexual behavior as a part of impulse control disorders are noted in 3.5% of PD patients using a dopamine agonist.^60^ A major setback of our study is that NMS-Quest does not evaluate this aspect. However, a recent study has shown improvement in impulse control disorders after STN DBS.^61^

***Miscellaneous***

Symptoms in the miscellaneous domain were less common in DBS group compared to controls. This is a heterogeneous group consisting of symptoms pertaining to thermoregulation (excessive sweating), pain (unexplained pains), drug effect (swelling of feet), weight changes and diplopia.^4^ On assessment of individual symptoms, complaints of pain were significantly less common in DBS group compared to medical therapy. Pain is a common complaint in PD and is worse in the “off” state.^62^ The etiology is varied and may be secondary to rigidity, dystonia or changes in pain perception. Recent study they found improvement in pain after bilateral STN DBS was almost universal and persisted for 2 years after the surgery.^63^ As is the case with many NMS, the effect may be secondary to improvement in motor functions but as DBS also seems to impact our sensory perception, it may be secondary to modulation of neural networks which may alter the central processing of pain.^64^

Our study compared the frequencies of various NMS among patients who have undergone DBS compared to those on medication alone. Reduction in dopaminergic dose is commonly seen after DBS and may contribute to the reduction in NMS such as orthostatic hypotension, cognitive impairment, depression and hallucinations, and gastrointestinal symptoms. Thus, DBS may help by directly stimulating the brainstem and by secondarily modifying medication.

***Pitfalls of study***

Our study used a simple tool in an attempt to identify the effect of STN DBS on the presence of NMS. However the NMS Quest only assesses the presence or absence, and does not evaluate the severity, of the NMS. NMS Quest does not cover certain areas such as gait, speech, dopamine dysregulation syndrome, and is subjective.

The second drawback is that we have used a case-control study which is fraught with selection biases. Moreover, we are using two groups of PD patients who are age and disease duration matched. Although the UPDRS ‘on’ scores were similar in both groups, the ‘off’ score was evaluated with the stimulator ‘on’ in the DBS group and hence does not give a clear picture of the disease severity. There is a possibility that the cases and controls have different disease severity and that itself might have contributed to the differences in NMS.

However, the only advantage of this design over a study comparing the symptoms before and after STN DBS in the same set of patients is that we were able to compare patients at the same time in the disease course. We also have undertaken the study considering the bilateral STN DBS group to be a homogenous one. Recent research has shown that surgical trajectory and final location of the electrode can significantly influence neuropsychological outcome and their effect on other NMS are not known.

## Conclusion

Overall NMS and symptoms in the domains of cardiovascular, gastrointestinal, sleep and miscellaneous were significantly less frequent, in patients who underwent bilateral STN DBS while sexual disturbances were significantly more frequent, when compared to patients on best medical treatment. This may be due to an either a direct effect of DBS or secondary to a reduction in medication. The impact on these scores on the functional and occupational status of the patients has still not been established. Further longitudinal cohort studies and randomized control studied are required to confirm these findings and compute the effect of DBS on various domains and functional outcome.

## Notes:

**How to cite this article: **Mridula KR, Borgohain R, Jabeen SA, Padmaja G, Srinivasarao Bandaru VCS, Ankathi A, et al. Comparison of frequencies of non motor symptoms in Indian Parkinson’s disease patients on medical management versus deep brain stimulation: A case-control study. Iran J Neurol 2015; 14(2): 86-93.
